# Impediments to and impact of checklists on performance of emergency interventions in primary care: an *in situ* simulation-based randomized controlled trial

**DOI:** 10.1080/02813432.2021.1973250

**Published:** 2021-09-13

**Authors:** Eric Dryver, Jeanette Knutsson, Ulf Ekelund, Anders Bergenfelz

**Affiliations:** a Department of Emergency and Internal Medicine, Skåne University Hospital at Lund, Lund, Sweden; b Department of Clinical Sciences at Lund (IKVL), Lund University, Lund, Sweden; c Practicum Clinical Skills Centre, Office for Medical Services, Region Skåne, Sweden

**Keywords:** Checklist, emergencies, patient safety, simulation training, primary health care

## Abstract

**Objective:**

Medical crises occur rather seldom in the primary care setting, but when they do, initial management impacts on morbidity and mortality. Factors that impede the performance of emergency interventions in primary care have not been studied through in-situ simulation. Checklists reportedly improve crisis management.

**Design:**

This randomized controlled trial evaluated emergency intervention performance during two scenarios (hypoglycemia-coma and anaphylaxis-cardiac arrest) simulated at primary care centers, and whether checklist access improved performance.

**Setting:**

Twenty-two primary care centers in Southern Sweden participated in the study.

**Subjects:**

A total of 347 personnel performed 100 simulations, 45 with and 55 without checklist access.

**Main outcome measures:**

Time and impediments to performance of five emergency interventions in each scenario.

**Results:**

On 28 of the 37 occasions when the adrenalin auto-injector was employed, the administration technique was incorrect. In 9 of 49 scenarios, teams had trouble locating the 30% glucose solution. Median time to supplemental oxygen administration during the first scenario was 186 s compared with 96 s during the second scenario (*p* < 0.001). Checklist access had no significant impact on time to performance of emergency interventions, aside from shorter time to adequate glucose or glucagon administration (median times 632 s with, 756 s without checklist access; *p* = 0.03).

**Conclusion:**

Unfamiliarity with local emergency equipment impedes the performance of emergency interventions during crises simulated in the primary care setting. Simply providing checklist access does not improve the performance of emergency interventions.

**KEY POINTS**
- Little is known about the factors that affect the performance of emergency interventions in the primary care setting.
- Unfamiliarity with local emergency equipment impedes the performance of emergency interventions during crises simulated in the primary care setting.
- Simply providing crisis checklist access does not improve the performance of emergency interventions in the primary care setting.

## Introduction

Medical crises are low-frequency, unexpected events where acute management impacts on patient morbidity. The archetypal medical crisis is cardiac arrest. Delay in the order of minutes in the initiation of chest compressions is associated with decreased survival [1]. In the setting of nonshockable rhythms, delay in the order of minutes between the initiation of chest compressions and the administration of epinephrine is independently associated with decreased survival [1]. In cases of anaphylaxis, delay in the administration of adrenalin is associated with fatal outcomes [2]. Severe hypoglycemia can lead to seizures, pulmonary aspiration, malignant ventricular arrhythmias [3] and, if sustained, irreversible brain damage [4]. Immediate administration of intravenous glucose or intramuscular glucagon is recommended [5].

While infrequent, cardiac arrests do occur in the primary care setting [6–8], and one study reported an incidence of severe hypoglycemia of 1.3% per year in type-1 diabetics and 0.9% per year in insulin-treated type-2 diabetics in the primary care setting [9]. There are but a handful of studies assessing the ability of personnel in the primary care setting to manage medical crises. One study of 53 general practitioners reported that 91% were unable to perform basic life support according to current guidelines [10]. More recent surveys suggest gaps in knowledge, training and equipment [6,8,11]. One systematic review [12] and several surveys (e.g. [13]) report deficiencies in knowledge relating to the recognition and management of anaphylaxis in the primary care setting. None of these studies identified impediments to performance through in-situ simulation.

Checklists are cognitive aids that augment memory and attention [14]. Cognitive aids such as checklists may help teams manage crises by palliating for the unfamiliarity and stressful nature of the situation [15,16]. Simulation, and in particular *in situ* simulation, is increasingly recognized as a technique to study rare events such as medical crises, identify latent errors and study the impact of new processes on health care delivery [17,18]. Two simulation-based studies showed that access to crisis checklists improved performance [19,20]. The aims of this *in situ* simulation-based study were to identify factors that impede the performance of a selection of emergency interventions in the primary care setting, and to evaluate whether the provision of crisis checklists improves performance.

## Materials and methods

### Context

The study was carried out within the framework of *in situ* simulation-based team-training offered by Practicum Clinical Skills Center to primary care centers in Region Skåne, the Southern Healthcare Region in Sweden. As of 2014, primary care center directors who requested *in situ* team-training were invited to combine, upon consent of participating personnel, *in situ* team-training with a trial evaluating the impact of access to three medical crisis checklists on time to performance of emergency interventions. The Regional Ethics Review Board of Lund approved the study (Dnr 2013/289).

### Lecture

Prior to simulation-training, participating personnel attended a 40-minute lecture that focused on the steps in a generic resuscitation algorithm as well as on communication, leadership and teamwork during crises. The lecture covered the appropriate use of adrenalin in anaphylaxis and cardiac arrest and the appropriate use of glucose in hypoglycemia. The lecture was delivered at the primary care center by the physician and the nurse running the simulation training. Participants randomized to checklist access were briefly presented with the three crisis checklists during the end of the lecture.

### Enrollment and allocation to checklist access and scenario

At the conclusion of the lecture, the personnel were informed of the option of combining in-situ team-training with a study, presented with the purpose and methodology of the study, and invited to participate. Personnel were informed that the simulations would be video-recorded if the personnel consented to participation in the study, that the focus was on team-performance and not on individual performance, that names and personal identification numbers would not be recorded, and that only study personnel would have access to the video recordings. If all or all but one of the participants attending the lecture consented to participate in the trial, participants filled out an informed consent formulary, received written information about the study, were included in the study and allocated to checklist access or not using a random sequence generated prior to study start. If two or more personnel did not want to participate in the study, team-training was performed outside of the context of the study.

When the number of participating personnel was large, personnel took part in only one of the two scenarios while other personnel were observers; when the number was smaller, some personnel took part in both scenarios. If the team-size dwindled to three participants or less when the second scenario was performed, instructors stepped in to provide manpower during the simulation and the simulation was excluded from the study.

### Simulations and feedback

Right after the lecture, two simulations (a hypoglycemia-coma scenario and then an anaphylaxis-arrest scenario) were carried out using a third-generation simulation manikin (SimMan 3 G, Laerdal®^)^ placed supine on a gurney in the primary care center's resuscitation room. This manikin featured a loudspeaker allowing one instructor to interact with the personnel and make snoring and vomiting sounds. The manikin's chest wall could simulate chest excursions associated with respiration, and featured electrodes to simulate cardiac electrical activity. The manikin's arms were equipped with rubber tubing to allow for simulated peripheral vein catheterization. A monitor was used to display pulse oximetry values, blood pressure and cardiac rhythm once the personnel had connected their pulse oximeter, blood pressure cuff and electrodes to the manikin, respectively. Personnel were instructed to use their center's equipment. Medications that personnel brought to the bedside were replaced by placebo, and cardiac arrest electrodes were replaced by cables connecting the primary care center's defibrillator to the manikin. Following each simulation, feedback was provided regarding emergency intervention performance and teamwork, and participants had the opportunity to practice using their local resuscitation equipment.

### Scenarios and emergency interventions

Emergency interventions were defined as physical or pharmacological measures that, when performed within the scope of minutes, arguably impact on morbidity and mortality and can be performed in the primary care setting. The training and trial centered around two scenarios each featuring five emergency interventions (Supplementary Appendix).

In the first scenario (hypoglycemia-coma), a 50-year-old unidentified man is found unconscious in the waiting room. Personnel are expected to perform five emergency interventions:

**Figure 1.**
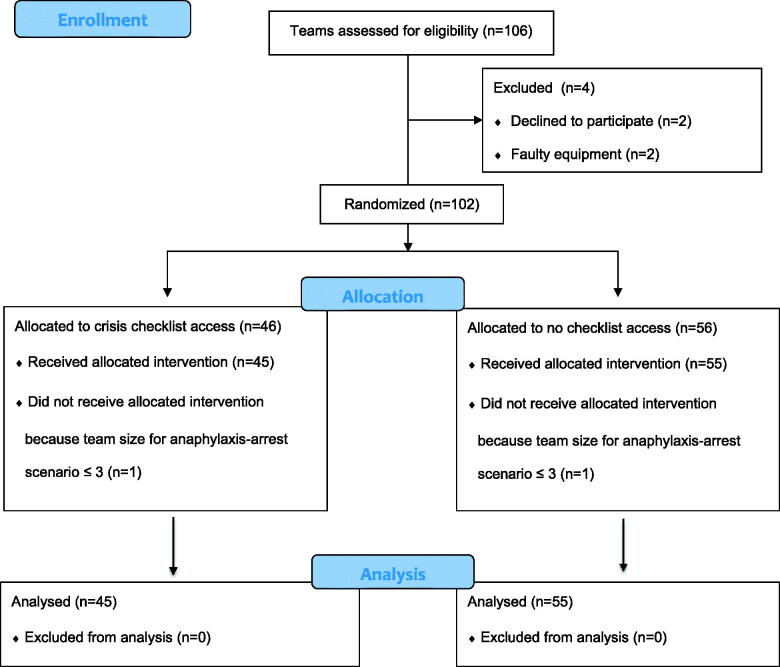
CONSORT flow diagram. CONSORT Flow Diagram itemizing the number of teams assessed for eligibility, excluded, randomized and analyzed.

- chin-lift or jaw-thrust to relieve upper airway obstruction due to loss of airway tone
- administration of supplemental oxygen via mask to address hypoxemia
- ventilation using either bag-mask or pocket-mask to address bradypnea
- intravenous administration of crystalloid to address hypotension
- intravenous administration of ≥20 ml of 30% glucose or intramuscular administration of glucagon to address severe hypoglycemia

In the second scenario (anaphylaxis-arrest), a 20-year-old patient presents to the primary care center after being stung by a wasp. Within minutes, the patient vomits and develops upper airway edema, stridor and hypotension rapidly progressing to cardiac arrest due to pulseless electrical activity (PEA). Personnel are expected to perform five emergency interventions:

**Figure 2.**
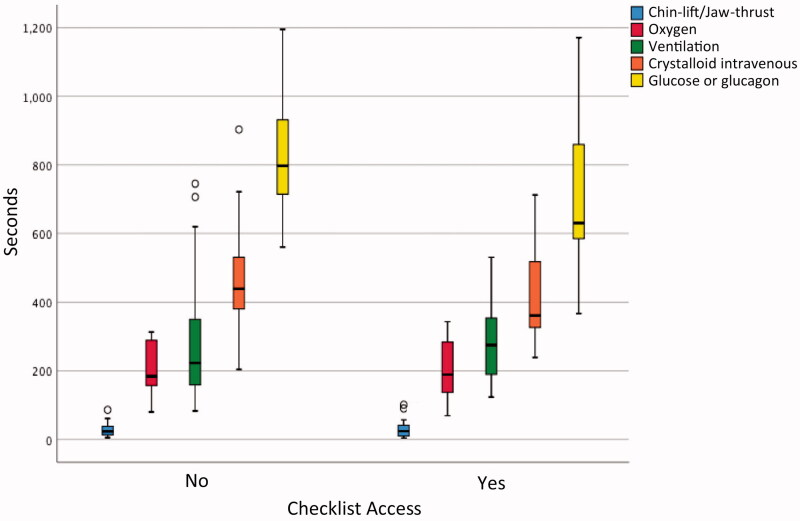
Time to performance of emergency interventions in the hypoglycemia-coma scenario. The Hypoglycemia-Coma scenario featured five emergency interventions. This figure illustrates the times (median, range and outliers) when these measures were performed from scenario start.

- intramuscular administration of 0.3–0.5 mg adrenalin following recognition of anaphylaxis
- administration of supplemental oxygen via mask to address hypoxemia
- intravenous administration of crystalloid to address hypotension
- chest compressions when the patient develops PEA
- subsequent intravenous administration of 1 mg adrenalin

### Checklists

Three crisis checklists (generic resuscitation, anaphylaxis, cardiac arrest) were developed by nurses and physicians over the course of several meetings (Appendix) (consider adding the word Supplementary for the sake of consistency). The nurses and physicians who developed the checklists all had experience working clinically in acute care settings and running simulated crises in the primary care setting. The generic resuscitation checklist listed emergency interventions to consider in the setting of managing a critically ill patient prior to establishing a diagnosis. It was based on the Swedish Society for Emergency Medicine's generic ABCDE algorithm [21] and adapted to the primary care setting. The anaphylaxis and cardiac arrest checklists were derived from European Resuscitation Guidelines [22,23] and adapted to the primary care setting. The checklists were printed on 70 cm by 105 cm posters. If the group had been randomized to checklist access, the checklist posters were mounted on the walls of the room and the two-sided checklist board was placed on the primary care center's crash cart. In addition, checklists were printed on a two-sided 28 cm by 42 cm rigid board and placed on the primary care center's crash cart.

### Outcome measures

The primary outcome measure was time to performance of the five emergency interventions in each scenario. Team performance was recorded using two video cameras. Time from scenario start until emergency intervention performance was determined by reviewing the video recordings. In the anaphylaxis-arrest scenario, scenario start was defined as the moment when the manikin vomits, develops stridor or complains of swelling of the upper airway, whichever came first; time to chest compressions and intravenous adrenalin was measured from onset of cardiac arrest. There was no pre-determined scenario end time; scenarios were terminated, as a rule, when the teams performed the fifth emergency intervention.

In order to perform an emergency intervention that is indicated, teams needed to:

- consider performing the intervention and recognize that it is indicated
- be able to locate and bring to the bedside required equipment/medications
- be able to perform the intervention correctly (e.g. connect oxygen tubing and open oxygen flow, deliver the correct medication dose).

Audio video footages was analyzed to identify and categorize occurrences that impeded the delivery of emergency interventions. After simulation training, a questionnaire evaluating the checklists was filled out by teams randomized to checklist access (Supplementary Appendix).

### Statistical analysis

Power calculations determined that 100 simulations were required to detect a decrease in the frequency of a lacking emergency intervention from 30 to 10% with an alpha risk of 5% and a statistical power of 80%. Chi^2^ test (linear-by-linear association) was used to analyze number of performed emergency interventions depending on checklist access. Mann–Whitney U-test was used to compare times to performance of emergency interventions. When analyzing the impact of checklists on time to intervention performance, missing times due to intervention not being performed were replaced:

- in the hypoglycemic coma scenario by total scenario duration.
- In the anaphylaxis-arrest scenario, for the first three interventions, by time until cardiac arrest; for the last two interventions, by time from cardiac arrest to scenario conclusion.

A two-tailed *p*-value of <0.05 was considered significant. Due to the exploratory nature of this study, no Bonferroni adjustments for multiple testing were made. A random sample of ten hypoglycemic coma and ten anaphylaxis-arrest scenarios was independently reviewed by a second investigator to determine inter-observer variability. Cohen's kappa was used to measure inter-rater reliability regarding whether emergency interventions were performed or not. Descriptive statistics were used to measure reliability of recorded times when emergency interventions were performed, start times and arrest time in the anaphylaxis arrest scenario. Statistical analyses were carried out using SPSS version 25.

## Results

Between January 2014 and June 2016, participation in the study was offered on 53 consecutive occasions at 22 primary care centers in Skåne. On one occasion, at least two personnel withheld consent to participate in the study, resulting in failure to enroll two teams (one for each scenario). On two occasions, the number of personnel had dwindled to three by the time the anaphylaxis-arrest scenario was carried out, and on one occasion the performance of both teams could not be analyzed due to failure of video recording. In total the study included 100 scenarios (51 hypoglycemic coma scenarios and 49 anaphylaxis-cardiac arrest scenarios) of which 45 were randomized to checklist access and 55 were randomized to no checklist access (Figure 1).

**Figure 3.**
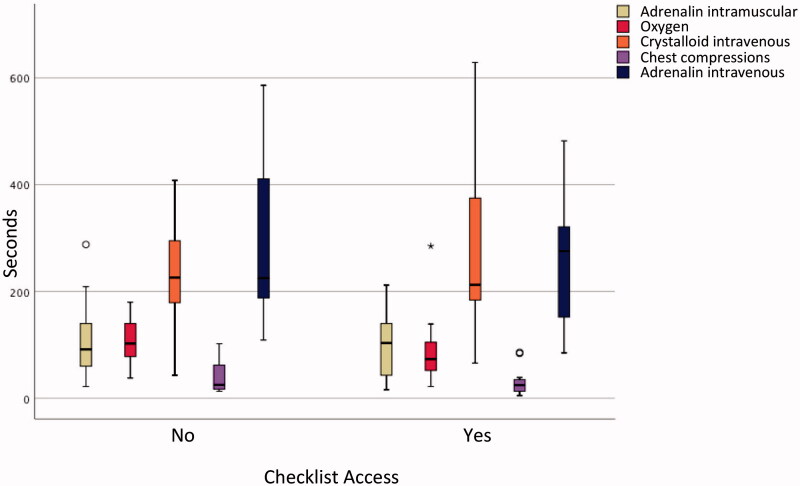
Time to performance of emergency interventions in the anaphylaxis-arrest scenario. The anaphylaxis-arrest scenario featured five emergency interventions. This figure illustrates the times (median, range and outliers) when intramuscular adrenalin, oxygen and intravenous crystalloid where administered from scenario start; and the times when chest compressions and 1 mg adrenalin intravenous were administered from cardiac arrest.

A total of 347 personnel participated in the study (294 women and 53 men). The median team size was four (range three to six). Ninety-eight teams featured at least one physician or resident and at least two nurses or one nurse and one nursing assistant; one team consisted of two physicians and one nurse, and one team consisted of two nurses and one medical student. The median personnel age was 47 years and the majority of personnel had over 10 years of working experience. There were no significant differences in size, composition, member age or experience between teams randomized to checklist access and those not (data not shown).

All five emergency interventions were performed in 63 scenarios; in 30 scenarios, four emergency interventions were performed and in seven scenarios, two or three emergency interventions were performed. Occurrences and times to performance of emergency interventions feature in Tables 1 and 2.

**Table 1.** Performance of emergency interventions.

Emergency intervention	Occurrences and times
Chin-lift or Jaw-Thrust to address upper airway obstruction	Performed in 100% of simulations (51/51) Performed within 60 seconds from simulation start in 90% of simulations (46/51)
Supplemental Oxygen *via* mask to address hypoxemia	Not considered in 5% of simulations (5/100) During the first scenario, difficulty connecting oxygen mask tubing to oxygen regulator and opening oxygen flow in 10% of simulations (5/51) First scenario: median time to performance 186 seconds (range 69–654) Second scenario: median time 96 seconds (range 1-285), significantly shorter than during first scenario (*p* < 0.001)
Ventilation *via* bag-valve-mask to address bradypnea	Not considered in 20% of simulations (10/51) Median time to ventilation from simulation start 227 seconds (range 52–745)
Crystalloid intravenous bolus to address hypotension	Not considered in 24% of simulations (24/100) No difficulties noted in finding or using required equipment
Glucose 30% 20 ml intravenous or glucagon intramuscular to address severe hypoglycemia	Not considered within 5 min of simulation start in 70% of simulations (36/51) Not considered within 10 min of simulation start in 20% of simulations (10/51) Trouble locating the glucose solution in 18% of simulations (9/49) Time between order to administer glucose/glucagon and administration exceeding 60 seconds in 60% of simulations (30/51) Inadequate initial glucose dose in 30% of simulations (14/49)
Chest compressions for cardiac arrest	Not considered within 30 seconds of onset of cardiac arrest in 50% of simulations (25/49) Not considered within 60 seconds of onset of cardiac arrest in 22% of simulations (11/49)
Adrenalin 1 mg intravenous bolus to address pulseless electrical activity	Intravenous adrenalin not considered in 8% of simulations (4/49) Trouble locating the adrenalin in 2% of simulations (1/49) Insufficient dose administered in 22% of simulations (11/49) Not administered as bolus in 2% of simulations (1/49)

This table provides, for each of seven emergency interventions, the frequency and/or timing when the interventions were performed and the frequency of occurrences that impeded the performance of the intervention, regardless of checklist access.

**Table 2.** Adrenalin administration in anaphylaxis.

Method	Number of occurrences among 49 simulations, *n* (%)
Auto-injector	Right side up and cap removed (correct): 9 (18) Right side up and cap not removed: 13 (27) Upside down and cap removed: 6 (12) Upside down and cap not removed: 1 (2) Prolonged attempts to unscrew the needle end: 6 (12) Left in the thigh for less than one second: 2 (4)
Adrenalin 1 mg/ml	Correct intramuscular administration: 8 (16) Administered as intravenous bolus: 1 (2)
No adrenalin administered	No intention to administer adrenalin: 2 (4) Unable to locate auto-injector or adrenalin 1 mg/ml: 1 (2)

This table itemizes the use of adrenalin in the setting of a patient with stridor, tongue swelling, hypoxemia and shock following a wasp bite. Intramuscular adrenalin was successfully delivered in 35% of simulations (17/49).

Access to crisis checklists had no impact on whether emergency interventions were carried out or not. Among teams with checklist access, fewer than five emergency interventions were performed in a third of all scenarios (16/45), incorrect adrenalin dose was administered during cardiac arrest in a fourth of all cases (6/22), and correct 30% glucose volume was not ordered in a sixth of scenarios (3/19). However, checklist access was associated with a significantly shorter time to adequate administration of glucose in the hypoglycemic coma scenario (median times 632 s with checklist, 756 s without checklist, *p* = 0.03) but did not shorten time to performance of the other nine emergency interventions (Figures 2 and 3).

Cohen's kappa pertaining to performance of 100 emergency interventions as evaluated on video was 0.81. Disagreement between investigators related to whether oxygen was adequately administered (two hypoglycemia scenarios) and whether ventilation was adequately performed (one hypoglycemia scenario). Recorded times for intervention performance, start times and arrest times differed by five seconds or less in 115 of 120 paired observations. In three scenarios, differences ranged from seven to nine seconds and in two from 20 to 23 s (both relating to crystalloid infusion). Differences were resolved through consensus and did not substantially alter the study results. On a Likert scale of 1 to 6, 90% of all participants agreed (i.e. gave a score of 5 or 6) with the statement ‘I would use the checklists if I got a similar case in reality’ (Supplementary Appendix).

## Discussion

### Principal findings

This multicenter *in situ* simulation-based study randomized teams working in primary care to access to three medical crisis checklists; the trial did not demonstrate an effect of checklists on the performance of emergency interventions, aside from a shorter time to administration of adequate amount of glucose or glucagon in the hypoglycemic coma scenario. Teams had difficulty connecting oxygen mask tubing to the oxygen regulator and opening oxygen flow in 10% of hypoxemia instances, failed to ventilate in 20% of bradypnea instances, had trouble locating concentrated glucose solution in 18% of hypoglycemia coma instances and delivered an inadequate initial dose in 29%, did not deliver adrenalin according to current guidelines in 35% of PEA instances, and failed to deliver intramuscular adrenalin in 65% of anaphylaxis instances. The adrenalin auto-injector was not used properly in 76% of instances. Median time to supplemental oxygen administration during the first scenario was 186 s compared with 96 s during the second scenario.

### Strengths and weaknesses

Health care delivery is influenced by the attributes of health care professionals, the nature of the tasks (e.g. administering an adrenalin auto-injector), the available tools and technology (e.g. the type of defibrillator), the physical environment (e.g. where the defibrillator is located), organizational aspects (e.g. who is entitled to use the defibrillator), and interactions between these factors [24]. Survey-based studies have a limited ability to detect latent errors, such as the inability to find and use actual equipment. Studies evaluating new tools in simulated centers with volunteer personnel may not necessarily reflect how tools perform when used by actual health care personnel within their own work environment. The major strength of this study was that the simulations were performed *in situ*, at over 20 primary care centers, by unselected health care personnel, within their own working environments, and having to locate their own equipment and medications. Our study results may not be generalizable to primary care centers where the composition of teams managing medical crises differs from the one in the present study.

This study was carried out within the framework of *in situ* simulation-based team-training. The study's main weakness is that the simulations were preceded by a lecture that covered generic resuscitation steps, doses of adrenalin therapy in anaphylaxis and cardiac arrest, and glucose and glucagon therapy in hypoglycemia. Study participants likely anticipated that cardiac arrest and anaphylaxis would occur in at least one of the scenarios, and in anticipation of the training sessions, personnel may have refreshed their knowledge of emergency medicine equipment. These factors likely improved performance during the simulations and detracted from the value of checklist access. However, despite all team-members having attended a lecture prior to simulation, 37% of teams failed to perform all five emergency interventions, and adrenalin was not administered according to current guidelines in 35% of simulated cardiac arrests. An ideal study would not have included a lecture prior to simulation, but it is unlikely that a simulation-based in-situ study of this magnitude could have been conducted in the Swedish healthcare setting outside of the context of team-training.

### Findings in relation to other studies

One study reported that, among 53 general practitioners, 91% were unable to perform basic life support according to current guidelines [10]. In the present study, personnel initiated chest compressions without delay, yet our data suggest that many teams struggled to perform bag-valve-mask ventilation. One survey reported that fewer than half of respondents were confident in being able to administer an adrenalin auto-injector pen [13]. The current study found that the auto-injector was not used correctly in 76% of instances.

A simulation-based study reported that access to surgical-crisis checklists decreased the rate of missed lifesaving processes of care from 23 to 6% [19]. Another study reported that access to checklists for intensive care unit emergency procedures improved the completion of critical treatment steps [20]. Both of these studies were conducted in simulated environments with volunteer participants, and the results may not necessarily reflect how checklists would affect the performance of personnel in their own working environments. In contrast, this *in situ* simulation-based study did not demonstrate that checklists impacted significantly on health care delivery.

### Meaning of the study

Checklists are simple tools and their success in decreasing catheter-related bloodstream infections in the intensive care unit [25] and morbidity and mortality following surgery [26] has led to increased interest in applying checklists to improve performance within other health care domains. A limited number of studies report that crisis checklists improve performance [19,20]. Crisis checklists presumably work by helping team-members consider emergency interventions and by providing information on intervention indications and performance.

There may be several reasons for why crisis checklists did not improve performance in this study. First, checklists may not be the appropriate tool to improve specific processes [27]. Checklists may not improve the performance of interventions that hinge on recognizing that a condition is present, nor on well-rehearsed responses to specific situations. Either of these explanations may account for why crisis checklist access had no impact on initiation of chest compressions.

Second, checklists cannot mitigate for difficulties in finding and using crisis equipment, which in this study may have masked a positive effect of checklists on team performance. Some teams struggled to use their own equipment, for example connecting oxygen mask to oxygen tank regulator and opening the oxygen flow. The fact that median time to oxygen administration was twice as long during the first scenario than during the second argues for technical difficulties and the benefit team-members derived from familiarizing themselves with their equipment during the first scenario and between scenarios. In 18% of scenarios, teams had trouble locating 30% glucose solution, and time between the order to administer glucose or glucagon and actual administration stretched up to 5 min. In particular, the adrenalin auto-injector was used incorrectly in 76% of instances, highlighting the technical challenges associated with using crisis equipment during a crisis.

Third, some sources emphasize the importance of providing personnel with specific instructions and training as to how and when to use checklists, and assigning the responsibility of ensuring that the checklist is systematically used to a team member [27,28]. Yet it is dubious that primary care centers can afford to invest significant resources in implementing regular crisis checklist training given that managing critical patients is not the focus of their mission. This study assessed whether the simple provision of checklists impacted on team-performance. Crisis checklists were but briefly presented to teams randomized to checklists during the lecture. Despite having access to crisis checklists, fewer than five emergency interventions were performed in 36% of simulations and an incorrect adrenalin dose was administered during cardiac arrest in 27% of simulations, arguing that the checklists were not used as intended. Checklist format may also have impacted negatively on their use, yet most team-members surveyed opined that the checklists were user-friendly (Supplementary Appendix).

## Conclusions

Ensuring that personnel working in primary care centers are and feel competent in performing emergency interventions is challenging given that medical crises rarely occur at their workplace. The results from this study suggest that unfamiliarity with emergency equipment is an important factor that impedes the performance of emergency interventions. Regularly recurring, short, locally run training sessions dedicated to locating and using emergency equipment may address this issue. In-situ simulation of medical crises in the primary care setting has been shown to increase the self-reported confidence and competence of personnel [29].

In-situ simulation may also be used to study the impact of new processes on observed health care delivery during medical emergencies [17,18]. This study showed no evidence that checklist access improved performance during simulated crises, yet the results also suggest that the checklists were not used as intended, perhaps due to their unfamiliarity. There was no evidence that checklist access impeded performance, and 90% of all participants expressed that they would use the checklists if they got a similar case in reality. Mounting large posters featuring crisis checklists on the walls of rooms dedicated to resuscitation may be justifiable and refreshing key algorithms could be integrated with regular equipment training. Yet this study suggests that doing so does not constitute a ‘quick fix’ to the challenges faced by primary care personnel when taking care of critically ill patients.
